# Effects of body weight and fiber sources on fiber digestibility and short chain fatty acid concentration in growing pigs

**DOI:** 10.5713/ajas.19.0713

**Published:** 2019-12-24

**Authors:** Jinbiao Zhao, Xuzhou Liu, Yi Zhang, Ling Liu, Junjun Wang, Shuai Zhang

**Affiliations:** 1 State Key Laboratory of Animal Nutrition, Ministry of Agriculture Feed Industry Centre, China Agricultural University, Beijing 100193, China; 2 Institute of Mycology/Engineering Research Center of Chinese Ministry of Education for Edible and Medicinal Fungi, Jilin Agricultural University, Changchun 130118, China; 3 Beijing E-Feed & E-Vet Cooperation, Beijing 100029, China

**Keywords:** Body Weight, Fiber Sources, Fiber Fermentation, Growing Pig, Short Chain Fatty Acids

## Abstract

**Objective:**

The study was conducted to determine the effects of body weight (BW) and fiber sources on nutrient digestibility, fiber fermentation and short chain fatty acids (SCFA) concentration in different intestinal segments of growing pigs fed high-fiber diets.

**Methods:**

Nine barrows with initial BW of 25.17±0.73 kg and 9 barrows with initial BW of 63.47±2.18 kg were allotted to a duplicate 9×2 Youden Square design with 3 dietary treatments and 2 periods. The dietary treatments were formulated with 3 different high-fiber ingredients: corn bran, sugar beet pulp, and soybean hulls, respectively. Each diet was fed to 3 barrows with different stage of BW in each period.

**Results:**

There were no differences in the apparent ileal digestibility (AID) of most nutrients between pigs at different BW stages. Pigs at 60 kg had greater (p<0.05) apparent total tract digestibility (ATTD) of total dietary fiber (TDF), soluble dietary fiber (SDF) and insoluble dietary fiber (IDF), and had greater (p<0.05) hindgut disappearance of IDF and cellulose than pigs at 25 kg. The acetate, propionate and total SCFA concentrations in ileal digesta and feces of pigs at 60 kg were greater (p<0.05) than those of pigs at 25 kg. In addition, fiber sources affected (p<0.05) the AID of gross energy (GE), organic matter (OM), ether extract (EE), crude protein, SDF and hemicellulose, the hindgut disappearance and ATTD of dietary fiber components, the lactate and propionate concentrations in ileal digesta and the butyrate, valerate and total SCFA concentrations in feces. There were interactions (p<0.05) between BW and fiber sources on the AID of GE, OM, EE, SDF, hemicellulose, the ATTD of EE, TDF, and IDF, and the hindgut disappearance of SDF and hemicellulose.

**Conclusion:**

Increasing BW mainly improved the digestibility of dietary fiber fractions, and the dietary fiber sources influenced the digestibility of almost all the dietary nutrients in growing pigs.

## INTRODUCTION

Traditionally, dietary fiber is resistant to the endogenous enzymatic digestion in the small intestine of pigs, but it can be fermented by bacteria in the hindgut of pigs to produce short chain fatty acids (SCFA) [1]. The SCFA is considered to exert a beneficial impact on the gut health of the host [2]. Recently, some studies have reported that dietary fiber fractions could also be fermented in the small intestine of pigs [3,4] because the fiber-degrading bacteria are observed to be present in the stomach and small intestine of pigs. In addition, many studies have demonstrated that the energy and nutrients digestibility in the high-fiber diets was affected by the fiber sources [5,6] due to their different chemical compositions and physical properties. However, there were only a few studies reporting the effect of fiber sources on nutrient digestibility in different digestive sites of pigs [4,7].

It is also known that the nutrient and energy digestibility of feed ingredients in swine diets could be affected by the body weight (BW) of pigs. The improved apparent total tract digestibility (ATTD) of nutrients and energy in diets was observed with the increased BW when adult sows and growing pigs were compared [8]. Moreover, Huang et al [9] reported that the apparent ileal digestibility (AID) of carbohydrates and the ATTD of dry matter (DM), ash, organic matter (OM), carbohydrates and gross energy (GE) in diets with the inclusion rate of wheat bran were significantly different when fed to growing pigs with different BWs. However, there is little knowledge about the interactive effects of BW and fiber sources on the nutrient digestibility in the small intestine and hindgut of pigs.

Corn bran (CB), sugar beet pulp (SBP), and soybean hulls (SH) are three crop co-products that are commonly used in swine diets, but these ingredients have different chemical compositions and physical characteristics of fiber fractions [6,10]. The ingredients of CB are rich in insoluble fiber fractions and contain a high content of hemicellulose [11], while SBP has more soluble fiber fractions [12]. SH contains a high content of insoluble fiber fractions, and also contains a high content of fermentable oligosaccharides and soluble fiber fractions [13]. No previous studies have focused on the interactive effects of BW and these three fiber sources on nutrient digestibility in different intestinal segments of growing pigs. Therefore, the objective of this study was to investigate effects of BW and fiber sources on fiber components digestibility and SCFA concentration in different intestinal segments of growing pigs fed diets containing CB, SBP, or SH.

## MATERIALS AND METHODS

The Laboratory Animal Welfare and Animal Experimental Ethical Inspection Committee in China Agricultural University (Beijing, CAU20150925-2) reviewed and approved all protocols used in this study. The animal trial was conducted at the Swine Research Unit of China Agricultural University (Beijing, China).

### Animals and housing

Nine Duroc×(Landrace×Yorkshire) barrows with initial BW of 25.17±0.73 kg and 9 barrows with initial BW of 63.47±2.18 kg were surgically fitted with a T-cannula in the distal ileum followed the protocol described by Stein et al [14]. Pigs were allowed a 15-day recovery period after surgery and a commercial corn-soybean meal diet was fed during this period. The chemical composition of corn-soybean meal was formulated to meet nutrient requirements for pigs recommended by NRC [15]. All pigs were housed in individual stainless-steel metabolism crates (1.4 m×0.7 m×0.6 m) equipped with a nipple drinker and a feeder. The room temperature was kept between 20°C to 25°C throughout the experiment.

### Experimental design

Barrows were allotted to a 9×2 Youden Square design with 3 dietary treatments and 2 periods, respectively. The dietary treatments included 3 different high-fiber ingredients (CB, SBP, or SH), which were the sole fiber source in each treatment diet. Each diet was fed to 3 barrows with different stage of BW in each period. The chemical compositions of CB, SBP, and SH were analyzed and presented in Table 1. The feed ingredients and analyzed chemical compositions of the three experimental diets are shown in Table 2 and Table 3, respectively. Each experimental diet was randomly assigned to 3 pigs at each BW stage in each period, and each period lasted for 15 days, including 10 days for diet adaptation, 3 days for fecal collection, and 2 days for digesta collection. Vitamins and minerals were supplied in all diets to meet or exceed the nutrient requirements for pigs recommended by NRC [15]. An amount of 3.0 g/kg chromic oxide was included in all diets as an indigestible index.

All pigs had *ad libitum* access to water, and were fed a daily amount of feed equivalent to 4% of their initial BW, which were divided into 2 equal meals provided at 0800 and 1600 h. The BW of each pig was recorded at the beginning of the experiment and at the end of each period to calculate the feed allowance.

### Sample collection

Fecal samples were collected on d 11 to d 13 of each period via grab-sampling and stored at −20°C. Digesta samples were collected on day 14 and 15 of each period. Collection of ileal digesta was initiated at 0800 h and ceased at 1800 h on each collection day into a plastic bag attached to the barrel of the cannula using a cable tie. The bags were removed when filled with digesta and then were stored at −20°C to prevent bacterial degradation of the samples. At the end of each period, digesta and fecal samples were thawed and mixed within pig and diet. A sub-sample was lyophilized and ground through a 1-mm screen and stored at −20°C prior to chemical analysis. Furthermore, ileal digesta was collected from the cannula directly and fresh fecal sample was collected immediately as soon as feces of pig appeared. Fresh fecal and ileal digesta samples were collected and immediately snap-frozen using liquid nitrogen, and stored at −80°C for the SCFA analysis.

### Chemical analyses

The DM (Method 934.01), crude protein (CP; Method 990.03), ether extract (EE; Method 920.39), ash (Method 942.05), soluble dietary fiber (SDF; Method 991.43), insoluble dietary fiber (IDF; Method 991.43), and chromium (Method 990.08) contents in diets, digesta and feces were determined according to the procedures of the Association of Official Analytical Chemists (AOAC) [16]. The OM content was calculated as the difference between DM and ash contents. The total dietary fiber (TDF) content was calculated as the sum of SDF and IDF contents. The neutral detergent fiber (NDF) and acid detergent fiber (ADF) contents were analyzed using fiber bags (Model F57, Ankom Technology, Macedon, NY, USA) and a fiber analyzer (ANKOM^200^ Fiber Analyzer, Ankom Technology, USA) after an adaptation of the procedures described by Van Soest et al [17]. The NDF content was determined using heat stable α-amylase and sodium sulphite without correction for insoluble ash. The content of acid detergent lignin (ADL) was also determined following the guidance of Ankom Technology (USA). The hemicellulose content was calculated as the difference between NDF and ADF contents, and the cellulose content was calculated as the difference between ADF and ADL contents. The GE in feces, diets and ingredients were measured using an Automatic Isoperibol Oxygen Bomb Calorimeter (Parr 1281 Calorimeter, Moline, IL, USA).

The amino acid (AA; Method 151 982.30) contents in ingredients, diets and digesta were analyzed according to the procedures of AOAC [16]. Specifically, samples were hydrolyzed with 6 *N* HCl at 110°C for 24 h and then analyzed for 15 AA using an Amino Acid Analyzer (Hitachi L-8900, Tokyo, Japan). Methionine and cystine were determined as methionine sulfone and cysteic acid after cold performic acid oxidation overnight and hydrolyzing with 7.5 *N* HCl at 110°C for 24 h using an Amino Acid Analyzer (Hitachi L-8800, Japan). Tryptophan was determined after LiOH hydrolysis for 22 h at 110°C using HPLC (Agilent 1200 Series, Santa Clara, CA, USA).

The concentrations of lactate and SCFA in ileal digesta and fecal samples were determined using a modified method of Porter and Murray [18]. Specifically, about 1.0 g sample was diluted with 2.0 mL of 0.10% HCl solution, kept on ice for 30 min, and then centrifuged at 12,000 g at 0°C for 15 min. A 1.0 mL of the supernatant was passed through a 0.20 mm Nylon Membrane Filter (Millipore, Bedford, OH, USA) and then 5.0 μL of the solution was injected into a gas chromatographic system (Agilent HP 6890 Series, USA).

### Calculations

The AID and ATTD of DM, GE, CP, EE, ADF, NDF, OM, cellulose, hemicellulose, TDF, SDF, IDF, and AA were calculated in all diets using the following equation. The hindgut disappearance of energy and nutrients was calculated as the difference between the AID and ATTD of dietary energy and nutrients.

$${\text{AD}}_{\text{nutrient}}=1-({\text{CN}}_{\text{digesta or feces}}/{\text{DN}}_{\text{diet}})\times ({\text{Cr}}_{\text{diet}}/{\text{Cr}}_{\text{digesta or feces}}),$$

where AD_nutrient_ is the AID or ATTD (%) of dietary energy and nutrients; CN_digesta or feces_ is the energy and nutrient levels in ileal digesta or feces (g/kg); DN_diet_ is the energy and nutrient level in diets (g/kg).

### Statistical analysis

The UNIVARIATE procedure of SAS 9.2 (SAS Inst. Inc., Cary, NC, USA) was used to check the normality of residuals and equal variances. Outliers were identified as any value that deviated from the treatment mean by ±3 times of standard deviation. No outliers were observed in this experiment. Data were then analyzed as a 3×2 factorial treatment arrangement using the GLIMMIX procedure of SAS 9.2. An individual pig was treated as the experimental unit. The statistical model included the fixed main effects of BW, fiber sources, and their interaction effects. Period was also included in the model as a random effect. Statistical differences were separated by Duncan’s multiple range test. The significance level was set at p<0.05, whereas 0.05≤p<0.10 was considered as a tendency.

## RESULTS

All pigs remained healthy and readily consumed their diets. Both feces and digesta samples were successfully collected from all pigs. At the beginning of the animal trial, the average BW of the pigs were 25.17±0.73 kg and 63.47±2.18 kg, and the average BW of the pigs at the end were 41.34±1.85 kg and 74.28±3.79 kg at two different growth phases. The information of the pig BW and the treatment allocation are presented in Supplementary Table 1.

### Effect of body weight and fiber sources on apparent ileal digestibility of dietary chemical constituents

There were no differences in the AID of dietary GE, DM, ash, EE, TDF, SDF, IDF, NDF, and hemicellulose between pigs at 2 different BW stages (Table 4). Pigs at 60 kg tended to (0.05< p<0.10) have greater AID of dietary OM, ADF, and cellulose compared to those at 25 kg. Pigs fed the CB diet showed greatest (p<0.05) AID of GE, DM, OM, EE, and ash but lowest (p<0.05) AID of SDF and hemicellulose compared to those fed the SBP and SH diets. In addition, there were interaction effects (p<0.05) on the AID of GE, OM, EE, SDF, and hemicellulose between BW and fiber sources. Pigs at 60 kg had greater (p<0.05) AID of dietary GE, OM, SDF, and hemicellulose when fed the SBP diet, and greater (p<0.05) AID of dietary GE, OM, and hemicellulose when fed the SH diet, and lower (p<0.05) AID of dietary hemicellulose when fed the CB diet compared with pigs at 25 kg (Supplementary Table 2). However, the AID of dietary CP and AA, except for glutamic acid, glycine, alanine, valine and proline, were lower (p<0.05) in pigs at 60 kg than those in pigs at 25 kg (Table 5). Moreover, the AID of arginine, histidine, lysine, methionine, and proline in pigs fed the SBP diet were greater (p<0.05) than those in pigs fed the CB and SH diets. There were also interaction effects on the AID of dietary CP and most AA between BW and fiber sources, except for histidine, leucine, alanine, cystine, glutamic acid, glycine, and proline.

### Effect of body weight and fiber sources on apparent total tract digestibility of dietary chemical constituents

Pigs at 60 kg had greater (p<0.05) ATTD of dietary TDF, SDF, and IDF compared with pigs at 25 kg (Table 6). In addition, pigs fed the CB diet showed lower (p<0.05) ATTD of all dietary nutrients, except for EE and ash compared with pigs fed the SBP and SH diets. There were interaction effects (p<0.05) on the ATTD of dietary EE, TDF, and IDF between BW and fiber sources. Pigs at 60 kg showed greater ATTD of TDF and IDF in SBP and SH diets compared with those at 25 kg, but BW did not affect the AID of fiber components when pigs were fed the CB diet (data not shown).

### Effect of body weight and fiber sources on hindgut disappearance of dietary chemical constituents

Pigs at 60 kg had greater (p<0.05) hindgut disappearance of dietary IDF and cellulose compared with pigs at 25 kg (Table 7). The hindgut disappearance of dietary GE, DM, OM, TDF, IDF, NDF, ADF, cellulose and hemicellulose in pigs fed the CB diet were lower (p<0.05) than those in pigs fed the SBP and SH diets. There were interaction effects (p<0.05) on the hindgut disappearance of SDF and hemicellulose between BW and fiber sources.

### Effect of body weight and fiber sources on SCFA concentrations in ileal digesta and feces of pigs

Pigs at 60 kg had greater (p<0.05) concentrations of lactate, acetate, propionate and total SCFA in the ileal digesta, and greater (p<0.05) concentrations of acetate, propionate, butyrate, valerate and total SCFA in feces compared with pigs at 25 kg (Table 8). The lactate concentration in ileal digesta of pigs fed the CB diet was greater (p<0.05) than that of pigs fed the SBP diets, while the butyrate, valerate and total SCFA contents in feces of pigs fed the SH diet was higher (p<0.05) than those pigs fed the CB and SBP diets. Furthermore, there were no interaction effects on SCFA concentrations, except for the fecal valerate content, between BW and fiber sources.

## DISCUSSION

### Interactive effects on fiber digestibility between body weight and fiber sources

In the present study, there were some interactions on the AID of GE, OM, SDF, hemicellulose and the ATTD of TDF and IDF between BW and dietary fiber sources. Pigs with greater BW showed increased AID of GE, DM, OM, SDF and hemicellulose when fed the SBP and SH diets, but BW did not affect the nutrient digestibility when the CB diet was fed. The different effects of BW on the AID of SDF and hemicellulose may be associated with the more fermentable non-starch polysaccharides in SBP and SH, such as pectins [19], resulting in greater AID of dietary GE and OM. Similarly, increased BW of pigs improved the ATTD of TDF and IDF in the SBP and SH diets, but had no positive effects on the ATTD of dietary fiber in the CB diet. Those results indicated that the effects of BW on dietary nutrient digestibility were affected by fiber sources.

### Effect of fiber sources on fiber digestibility and SCFA concentration in different digestive sites of pigs

In the present study, the AID of dietary GE and DM for pigs fed the CB diet were greater than those fed the SBP and SH diets. The higher AID of GE and OM in CB diet was mainly caused by a greater EE digestibility, which is relative to amounts of EE in CB caused by processing technology. The AID of dietary SDF and hemicellulose in CB diet were lower compared with those in SH and SBP diets in this study, which may be caused by more fermentable SDF contents in SBP and SH diets. The above contradiction between greater energy digestibility and lower SDF digestibility in the CB diet could be explained by the small proportion of SDF in CB, resulting in a lower contribution of SDF digestibility to the energy utilization efficiency. In addition, the AID of dietary CP and most AA were affected by the dietary fiber sources. A possible explanation for the low AA digestibility in the CB diet was that IDF in CB increased the evacuation rate and decreased the retention time of digesta in the gut. Besides, another possible reason for the effect of different fiber sources on AA digestibility was the difference in endogenous nitrogen losses in pig intestine [20].

In this study, the ATTD of dietary GE, DM, OM, TDF, SDF, IDF, NDF, ADF, cellulose, and hemicellulose in pigs fed the SBP and SH diets were greater than those fed the CB diet, which was in accordance with the results from Chabeauti et al [21], who reported the ATTD of non-starch polysaccharides in growing pigs to be 16.3% for wheat straw, 43.5% for wheat bran, 69.5% for SBP, and 79.1% for SH. The lower ATTD of nutrients in CB diet may be caused by its high IDF content, which is difficult to be fermented by gut bacteria and could speed up the evacuation rate of digesta in the gut of pigs [12]. Moreover, the ATTD of dietary EE in pigs fed the CB diet was greater than those fed the SBP and SH diets, which may be caused by the high endogenous losses of EE in SBP and SH groups, resulting from dietary fiber fermentation to produce SCFA.

In the present study, there were differences in the hindgut disappearance of all dietary nutrients we tested, except for EE and SDF, among pigs fed the CB, SBP, and SH diets. The result was associated with the greater SDF concentration in SBP and SH diets and its higher fermentation ability in pigs. The result of negative values for the hindgut disappearance of dietary EE was consistent with the previous study [22], which is most likely a consequence of fatty acids synthesis in the hindgut, because the presence of carbohydrates in the hindgut allows microbes to synthesize fatty acids.

Pigs fed the CB diet had greater lactate concentration in the ileal digesta, but lower content of butyrate, valerate and total SCFA in the feces compared with those fed the SBP and SH diets. The lower concentration of SCFA in the CB treatment may be caused by the high IDF content in CB, which is consistent with the results of lower fiber fermentation in CB diet. On the other hand, SBP and SH are rich in SDF fraction that could be largely fermented to produce SCFA in the hindgut of pigs. However, pigs fed the SBP diet demonstrated lower nutrient digestibility compared to those fed the SH diet, while SBP had greater proportion of SDF compared to SH, which could be ascribed to the higher lignin content in SBP that is difficult to be fermented by gut microbiota. Moreover, the present study showed that pigs fed the SH diet had the greatest SCFA concentration in the feces, which was consistent with the results reported by Freire et al. [23], who compared the effects of the inclusion rate of 20% wheat bran, SBP, SH, and alfalfa meal on total SCFA concentration in the cecum of weanling pigs, and found that dietary SH inclusion increased the total SCFA concentrations by 11.2%, 30.5%, and 27.2% as compared with diets containing wheat bran, SBP and alfalfa meal, respectively.

### Effects of body weight on fiber digestibility and SCFA concentration in different digestive sites of pigs

There were no differences in the AID of dietary nutrients between pigs at 60 kg and pigs at 25 kg in our study. These results were consistent with the previous study, which reported that AID of most nutrients and energy in high-fiber diets with wheat bran supplementation were not affected by BW except for carbohydrates [9]. The explanation for the previous result was that the small intestine is relatively fully developed at 20 kg, while the large intestine keeps developing until 150 kg in pigs. Although there were no significant differences in the apparent nutrient digestibility in pigs at different BW, an increased SCFA concentration in the ileal digesta of pigs at 60 kg was observed, which may be associated with the trend of improved AID of dietary ADF and cellulose [24]. However, a study showed greater capacity of sows to digest fibrous components compared to growing pigs, and it was shown that sows could degrade a larger part of the dietary fiber in the small intestine than growing pigs [25]. These difference in the energy and nutrient digestibility with different BWs depends on the dietary fiber sources, which was demonstrated by our results through the interaction effects on some nutrient digestibility between BW and fiber sources. Furthermore, pigs at 60 kg had significantly lower AID of dietary CP and most AA compared with pigs at 25 kg in the present study, which was consistent with the previous report that the AID of CP and AA was significantly influenced by BW with in general around 1%-unit higher at low BW [26].

Pigs at 60 kg also had greater ATTD and hindgut disappearance of dietary TDF, IDF, and cellulose compared with pigs at 25 kg in the current study, resulting in the improved SCFA concentration in the feces. Huang et al [9] reported that the ATTD of DM, ash, OM, carbohydrates and GE in diets with wheat bran supplementation were significantly different when fed to pigs at different stages of BW. The ATTD of nutrients and energy in diets are improved with increased BW when adult sows and growing pigs are compared [8]. For weaned pigs, increased BW of pigs improved the ATTD of all dietary components except for NDF compared to pigs at three weeks post-weaning [27]. The main reason for the results that heavier pigs showed greater ileal digestibility of chemical components is that pigs with heavier BW have more developed and larger gastro-intestinal tract, slower digesta transit time, higher cellulolytic activity and increased fermentation capacity of microflora [28]. However, Le Goff and Noblet [29] reported that the apparent *in vivo* digestibility of dietary energy and most nutrients increased with BW of pigs, but they indicated that the greater capacity of heavy pigs or adult sows to digest dietary fiber is not due to an increased intrinsic ability of the microbial flora to degrade dietary fiber.

A previous study also showed that sows have a higher capacity to digest insoluble non-starch polysaccharides, whereas no difference in the digestibility of soluble non-starch polysaccharides was found between growing pigs and sows [30], which was consistent with the result in the present study. It may be caused by the high fermentable ability of dietary SDF in the small intestine of pigs. Overall, the BW of pig could affect the digestibility of dietary chemical constituents, especially nutrients digestibility in the hindgut of pig.

## CONCLUSION

High-fiber ingredients with different physicochemical properties had different effects on energy and nutrients digestibility, and SCFA concentration in the foregut and hindgut of pigs. The effect of pig BW on dietary energy and nutrients digestibility was influenced by dietary fiber sources. Therefore, it is necessary to take the dietary fiber sources into consideration when formulating diets for pigs at different growing stages.

## Figures and Tables

**Table 1:** The analyzed chemical compositions in corn bran, sugar beet pulp and soybean hulls (g/kg, dry matter basis)1)

Items	Corn bran	Sugar beet pulp	Soybean hulls
Gross energy (MJ/kg)	187.9	186.8	177.1
Crude protein	161.2	114.7	220.9
Ash	27.3	29.9	68.7
Organic matter	973.0	969.2	931.8
Ether extract	42.8	7.2	32.5
Neutral detergent fiber	565.1	595.1	678.7
Acid detergent fiber	176.1	244.3	488.5
Acid detergent lignin	24.0	69.2	19.1
Cellulose	152.2	175.1	469.4
Hemicellulose	389.0	350.8	190.2
Total dietary fiber	652.6	784.1	738.8
Soluble dietary fiber	75.0	310.2	148.6
Insoluble dietary fiber	577.6	474.0	590.1
Calcium	2.2	4.5	5.4
Phosphorus	2.2	1.8	1.9
Indispensable amino acids			
Arginine	9.0	8.6	13.5
Histidine	5.3	3.6	5.5
Leucine	14.5	7.1	15.3
Isoleucine	5.9	4.3	9.0
Lysine	7.7	9.0	14.8
Methionine	2.6	5.1	3.7
Phenylalanine	7.3	3.8	9.4
Threonine	6.8	5.2	8.7
Tryptophan	2.5	1.1	1.9
Valine	8.2	6.9	10.5
Dispensable amino acids			
Alanine	9.0	7.0	10.1
Aspartic acid	12.9	11.1	23.2
Cystine	2.9	1.3	4.4
Glutamic acid	30.2	29.2	33.2
Glycine	7.6	5.6	12.2
Proline	14.1	12.9	11.3
Serine	8.4	6.8	11.9
Tyrosine	3.9	5.6	5.6

1) All data are the results of chemical analysis conducted in duplicate.

**Table 2:** The composition (g/kg, as-is basis) of the experimental diets

Items	Diets		

	Corn bran	Sugar beet pulp	Soybean hulls
Ingredients			
Corn starch	446.0	444.5	425.9
Soy protein isolated	140.0	140.0	140.0
Corn bran	240.0	-	-
Sugar beet pulp	-	240.0	-
Soybean hulls	-	-	240.0
Soy oil	30.0	30.0	30.0
Sucrose	100.0	100.0	120.0
Limestone	5.5	-	-
Dicalcium phosphate	16.0	22.0	21.0
L-lysine-HCl	3.5	3.5	3.2
DL-methionine	1.0	1.5	1.4
L-threonine	1.5	2.0	2.0
Cr_2_O_3_	3.0	3.0	3.0
NaCl	4.5	4.5	4.5
K_2_CO_3_	3.0	3.0	3.0
MgO	1.0	1.0	1.0
Premix1)	5.0	5.0	5.0
Total	1,000	1,000	1,000

1) Premix provided the following quantities per kilogram of the complete feed for growing pigs: vitamin A, 5,512 IU; vitamin D_3_, 2,200 IU; vitamin E, 64 IU; vitamin K_3_, 2.2 mg; vitamin B_12_, 27.6 μg; riboflavin, 5.5 mg; pantothenic acid, 13.8 mg; niacin, 30.3 mg; choline chloride, 551 mg; Mn, 40 mg (MnO); Fe, 100 mg (FeSO_4_·H_2_O); Zn, 100 mg (ZnO); Cu, 100 mg (CuSO_4_·5H_2_O); I, 0.3 mg (CaI_2_); Se, 0.3 mg (Na_2_SeO_3_).

**Table 3:** The analyzed chemical compositions of the experimental diets (g/kg, as-is basis)1)

Items	Diets		

	Corn bran	Sugar beet pulp	Soybean hulls
Gross energy (MJ/kg)	16.98	16.67	16.81
Dry matter	919.1	917.1	918.5
Crude protein	122.3	126.2	123.4
Ether extract	16.8	9.4	14.6
Ash	39.5	44.9	44.6
Organic matter	878.6	872.2	873.9
Total dietary fiber	154.9	163.3	159.7
Soluble dietary fiber	16.3	60.6	28.4
Insoluble dietary fiber	138.6	102.7	131.3
Neutral detergent fiber	129.9	134.8	155.1
Acid detergent fiber	42.6	54.7	99.2
Cellulose	37.1	39.8	95.0
Hemicellulose	87.3	80.1	55.9
Indispensable amino acids			
Arginine	12.5	14.6	13.7
Histidine	3.8	4.8	5.3
Leucine	10.9	11.7	12.7
Isoleucine	5.9	7.4	7.5
Lysine	12.1	14.7	11.9
Methionine	4.1	3.3	4.2
Phenylalanine	6.3	7.1	8.6
Threonine	7.4	8.4	8.6
Tryptophan	2.5	3.0	3.2
Valine	2.1	1.3	2.0
Dispensable amino acids			
Alanine	4.7	5.5	5.9
Aspartic acid	13.5	17.1	18.6
Cystine	13.5	17.2	18.8
Glutamic acid	23.1	25.7	28.3
Glycine	5.1	6.1	7.9
Proline	11.6	12.7	11.5
Serine	5.9	7.2	8.1
Tyrosine	3.1	3.9	4.0

1) All data are the results of chemical analysis conducted in duplicate.

**Table 4:** Effects of pig body weight and fiber sources on the apparent ileal digestibility (%) of dietary nutrients1)

Items	Body weight		Diets			SEM	p-value		
		
	60 kg	25 kg	CB	SBP	SH		Weight	Source	Interaction
GE	72.27	71.38	73.49^a^	71.16^b^	70.31^b^	1.37	0.432	0.029	0.023
DM	69.38	67.85	71.13^a^	67.03^b^	67.19^b^	1.25	0.154	<0.001	0.068
Ash	17.01	19.73	24.29^a^	13.65^b^	17.17^ab^	4.89	0.111	<0.001	0.314
OM	72.61	70.85	73.80^a^	71.65^ab^	69.74^b^	1.11	0.064	<0.001	<0.001
EE	79.26	73.67	78.89^a^	68.59^b^	71.92^ab^	6.37	0.293	0.014	<0.001
TDF	8.92	8.41	2.09	15.84	8.05	5.52	0.663	0.094	0.323
SDF	27.85	22.67	9.68^c^	21.86^b^	44.25^a^	9.81	0.529	<0.001	<0.001
IDF	2.66	3.00	1.74	5.18	1.57	4.34	0.809	0.723	0.193
NDF	14.95	15.97	15.01	17.72	13.64	3.72	0.740	0.552	0.068
ADF	4.51	4.31	4.40	6.08	2.75	4.27	0.073	0.188	0.894
Cellulose	3.85	5.75	4.51	7.21	2.68	4.27	0.064	0.091	0.872
Hemicellulose	34.66	30.06	19.10^b^	38.94^a^	39.03^a^	4.37	0.210	<0.001	0.014

1) Data represent least square means (n = 6), and individual pig was treated as the experimental unit.

CB, corn bran; SBP, sugar beet pulp; SH, soybean hulls; SEM, standard error of the mean; GE, gross energy; DM, dry matter; OM, organic matter; EE, ether extract; TDF, total dietary fiber; SDF, soluble dietary fiber; IDF, insoluble dietary fiber; NDF, neutral detergent fiber; ADF, acid detergent fiber.

a–c Means with different superscript in the same row of body weight or diets are significantly different.

**Table 5:** Effects of pig body weight and fiber sources on the apparent ileal digestibility coefficient of amino acids1)

Items	Body weight		Diets			SEM	p-value		
		
	60 kg	25 kg	CB	SBP	SH		Weight	Source	Interaction
Crude protein	83.02^B^	86.19^A^	83.52^b^	84.25^ab^	86.05^a^	2.01	<0.001	0.022	<0.001
Indispensable amino acids									
Arginine	86.32^B^	92.00^A^	89.53^ab^	90.14^a^	87.80^b^	2.4	<0.001	<0.001	0.030
Histidine	83.44^B^	89.31^A^	85.04^b^	87.70^a^	86.38^ab^	1.54	<0.001	0.041	0.374
Isoleucine	84.96^B^	90.83^A^	86.95	88.44	88.29	1.32	<0.001	0.064	0.028
Leucine	84.22^B^	89.12^A^	85.53	87.80	86.69	1.26	0.023	0.079	0.174
Lysine	86.73^B^	92.08^A^	87.67^b^	91.81^a^	88.73^ab^	1.09	<0.001	<0.001	<0.001
Methionine	74.86^B^	78.74^A^	71.08^b^	86.31^a^	73.01^b^	2.46	<0.001	<0.001	<0.001
Phenylalanine	85.65^B^	89.87^A^	86.78	88.00	88.50	1.31	<0.001	0.059	0.042
Threonine	78.34^B^	88.34^A^	82.74	83.36	83.91	1.15	<0.001	0.073	0.014
Valine	87.21	83.80	85.90	85.53	85.10	1.15	0.154	0.611	0.020
Dispensable amino acids									
Alanine	86.20	86.31	83.59	89.21	85.96	3.74	0.932	0.157	0.271
Aspartic acid	84.46^B^	90.11^A^	86.58	88.26	87.02	1.27	<0.001	0.063	<0.001
Cystine	76.68^B^	88.66^A^	80.57^b^	82.80^ab^	84.64^a^	1.67	<0.001	<0.001	0.133
Glutamic acid	88.70	92.47	90.52	90.96	90.28	1.92	0.324	0.133	0.112
Glycine	83.40	83.19	87.76	78.52	83.59	5.98	0.910	0.240	0.758
Proline	69.09	64.15	68.27^ab^	77.37^a^	54.22^b^	8.25	0.667	0.031	0.143
Serine	76.37^B^	86.75^A^	81.75	82.80	80.12	1.36	<0.001	0.109	<0.001
Tyrosine	80.40^B^	82.50^A^	80.26^b^	80.50^b^	83.59^a^	1.60	<0.001	0.024	<0.001

1) Data represent least square means (n = 6), and individual pig was treated as the experimental unit.

CB, corn bran; SBP, sugar beet pulp; SH, soybean hulls; SEM, standard error of the mean.

A,B, a,b Means with different superscript in the same row of body weight or diets are significantly different.

**Table 6:** Effects of pig body weight and fiber sources on the apparent total tract digestibility (%) of dietary nutrients1)

Items	Body weight		Diets			SEM	p-value		
		
	60 kg	25 kg	CB	SBP	SH		Weight	Source	Interaction
GE	86.64	85.95	82.69^c^	89.50^a^	86.69^b^	0.72	0.263	<0.001	0.421
DM	86.04	85.31	81.90^c^	88.99^a^	86.14^b^	0.76	0.242	<0.001	0.294
Ash	45.47^B^	50.62^A^	49.41^a^	42.85^b^	51.88^a^	2.42	0.020	<0.001	0.058
OM	88.04	87.13	83.38^c^	91.49^a^	87.90^b^	0.69	0.121	<0.001	0.410
EE	43.19	36.01	46.71^a^	32.78^b^	39.30^b^	4.76	0.079	<0.001	<0.001
TDF	58.39^A^	51.21^B^	33.22^b^	69.21^a^	67.97^a^	7.22	0.037	<0.001	<0.001
SDF	82.74^A^	74.62^B^	58.27^b^	90.13^a^	87.64^a^	9.63	<0.001	<0.001	0.334
IDF	52.99^A^	47.11^B^	28.92^b^	57.53^a^	63.69^a^	8.15	0.020	<0.001	<0.001
NDF	58.22	60.03	40.17^b^	70.28^a^	66.92^a^	2.96	0.463	<0.001	0.211
ADF	51.95	50.87	30.39^b^	64.80^a^	59.05^a^	3.82	0.732	<0.001	0.510
Cellulose	59.87	56.94	33.14^c^	80.57^a^	61.49^b^	4.06	0.390	<0.001	0.592
Hemicellulose	68.09	67.56	43.13^b^	75.81^a^	77.03^a^	2.53	0.871	<0.001	0.384

1) Data represent least square means (n = 6), and individual pig was treated as the experimental unit.

CB, corn bran; SBP, sugar beet pulp; SH, soybean hulls; SEM, standard error of the mean; GE, gross energy; DM, dry matter; OM, organic matter; EE, ether extract; TDF, total dietary fiber; SDF, soluble dietary fiber; IDF, insoluble dietary fiber; NDF, neutral detergent fiber; ADF, acid detergent fiber.

A,B, a–c Means with different superscript in the same row of body weight or diets are significantly different.

**Table 7:** Effects of pig body weight and fiber sources on the hindgut disappearance (%) of dietary nutrients1)

Items	Body weight		Diets			SEM	p-value		
		
	60 kg	25 kg	CB	SBP	SH		Weight	Source	Interaction
GE	14.37	14.58	8.70^b^	18.34^a^	16.38^a^	1.61	0.881	<0.001	0.164
DM	16.66	17.46	10.27^c^	21.96^a^	18.96^b^	1.43	0.504	<0.001	0.203
Ash	28.46	30.89	25.12	29.21	34.71	5.61	0.742	0.122	0.520
OM	15.43	16.28	9.59^b^	19.83^a^	18.15^a^	1.25	0.420	<0.001	0.087
EE	−50.27	−37.66	−32.18	−57.10	−32.62	7.83	0.133	0.064	0.231
TDF	49.47	42.81	31.13b	53.37^a^	59.92^a^	6.54	0.062	<0.001	0.063
SDF	54.88	51.94	48.59	68.26	43.39	8.81	0.231	0.312	<0.001
IDF	50.33^A^	44.12^B^	27.19^b^	52.36^a^	62.12^a^	5.9	0.012	<0.001	0.222
NDF	43.26	44.06	25.15^b^	52.56^a^	53.28^a^	4.29	0.819	<0.001	0.448
ADF	47.45	46.56	25.99^b^	58.72^a^	56.30^a^	5.58	0.110	<0.001	0.842
Cellulose	56.02^A^	51.19^B^	28.64^c^	73.36^a^	58.81^b^	5.72	0.041	<0.001	0.664
Hemicellulose	33.43	37.50	24.03^b^	36.86^a^	38.00^a^	4.19	0.806	<0.001	0.041

1) Data represent least square means (n = 6), and individual pig was treated as the experimental unit.

CB, corn bran; SBP, sugar beet pulp; SH, soybean hulls; SEM, standard error of the mean; GE, gross energy; DM, dry matter; OM, organic matter; EE, ether extract; TDF, total dietary fiber; SDF, soluble dietary fiber; IDF, insoluble dietary fiber; NDF, neutral detergent fiber; ADF, acid detergent fiber.

A,B, a–c Means with different superscript in the same row of body weight or diets are significantly different.

**Table 8:** Effects of pig body weight and fiber sources on short chain fatty acids (mg/g) concentration in the fresh ileal digesta and feces of pigs1)

Items (mg/g)	Body weight		Diets			SEM	p-value		
		
	60 kg	25 kg	CB	SBP	SH		Weight	Source	Interaction
Feces									
Lactate	0.23	0.62	0.33	0.41	0.55	0.07	0.152	0.194	0.274
Acetate	10.32^A^	8.91^B^	8.06	9.52	11.27	0.93	0.011	0.152	0.322
Propionate	6.69^A^	4.84^B^	4.65	4.27	8.38	0.71	0.013	0.111	0.257
Isobutyrate	0.66	0.36	0.37	0.78	0.36	0.16	0.718	0.310	0.658
Butyrate	4.01^A^	2.95^B^	2.17^c^	3.31^b^	4.96^a^	0.46	0.014	0.008	0.082
Isovalerate	1.52	0.76	0.54	0.99	1.89	0.19	0.653	0.124	0.464
Valerate	1.47^A^	1.07^B^	0.49^b^	0.96^b^	2.36^a^	0.21	0.008	0.012	0.023
Total SCFAs	24.91^A^	19.74^B^	16.80^c^	20.35^b^	29.83^a^	1.29	0.006	0.011	0.241
Ileal digesta									
Lactate	3.45^A^	2.41^B^	3.77^a^	2.11^b^	2.90^ab^	0.93	0.031	0.014	0.391
Acetate	5.87^A^	2.83^B^	3.88	4.35	4.83	0.51	0.012	0.653	0.245
Propionate	1.08^A^	0.92^B^	0.60^b^	0.81^b^	1.60^a^	0.21	0.010	0.011	0.122
Isobutyrate	0.00	0.00	0.00	0.00	0.11	0.05	0.884	0.402	0.893
Butyrate	0.25	0.17	0.23	0.21	0.20	0.09	0.716	0.560	0.786
Total SCFAs	11.55^A^	6.54^B^	9.25	8.07	9.82	1.21	0.008	0.132	0.354

1) Data represent least square means (n = 6), and individual pig was treated as the experimental unit.

CB, corn bran; SBP, sugar beet pulp; SH, soybean hulls; SEM, standard error of the mean; SCFAs, short chain fatty acids.

Isobutyrate, isovalerate and valerate in the ileal digesta were not determined.

A,B, a–c Means with different superscript in the same row of body weight or diets are significantly different.
