# Supplementary material for: Effects of body weight and fiber sources on fiber digestibility and short chain fatty acid concentration in growing pigs
Source: Asian-Australas J Anim Sci. 2019 Dec 24;33(12):1975–84. doi: 10.5713/ajas.19.0713 (PMC7649408; doi:10.5713/ajas.19.0713)
Supplement: Supplementary file 1 [file ajas-19-0713-suppl.pdf]

Effects of body weight and fiber source on dietary fiber digestibility and short chain fatty acid concentration in growing pig

Supplementary Table 1-1. The body weight (BW) and feed allowance for the pigs of 25 kg

Pig number	Initial BW, kg	Diets	Feed allowance, kg/d	Final BW, kg
Period 1				
1	23	CB	0.92	32.5
2	23.5	SBP	0.94	33.15
3	24	SH	0.96	35.1
4	24.5	SH	0.98	29.9
5	25	SBP	1.00	30.55
6	25.5	CB	1.02	37.05
7	25.5	CB	1.02	31.2
8	27	SBP	1.08	31.85
9	28.5	SH	1.14	33.15
Period 2				
1	32.5	SBP	1.30	39.25
2	33.15	SH	1.33	40.96
3	35.1	CB	1.40	40.11
4	29.9	CB	1.20	40.96
5	30.55	SH	1.22	38.40
6	37.05	SBP	1.48	41.81
7	31.2	SBP	1.25	42.67
8	31.85	SH	1.27	42.67
9	33.15	CB	1.33	45.23

CB, corn bran; SBP, sugar beet pulp; SH, soybean hulls.

Supplementary Table 1-2. The body weight (BW) and feed allowance for the pigs of 60 kg

Pig number	Initial BW, kg	Diets	Feed allowance, kg/d	Final BW, kg
Period 1				
1	56.54	CB	2.26	62.50
2	60.79	SBP	2.43	66.44
3	61.91	SH	2.48	68.65
4	62.26	SH	2.49	66.00
5	62.73	SBP	2.51	68.98
6	65.28	CB	2.61	70.50
7	65.29	CB	2.61	72.21
8	66.29	SBP	2.65	71.89
9	70.13	SH	2.81	76.20
Period 2				
1	62.50	SBP	2.50	70.66
2	66.44	SH	2.66	73.73
3	68.65	CB	2.75	72.19
4	66.00	CB	2.64	69.11
5	68.98	SH	2.76	73.00
6	70.50	SBP	2.82	75.26
7	72.21	SBP	2.89	76.80
8	71.89	SH	2.88	76.80
9	76.20	CB	3.05	81.00

CB, corn bran; SBP, sugar beet pulp; SH, soybean hulls.

Supplementary Table 2. Effect of body weight on the apparent ileal digestibility (%) of GE, OM, SDF and hemicellulose in diets with different fiber sources.

	Diets		
Items	CB	SBP	SH
GE			
60 kg	72.04	72.89[a]	71.87[a]
25 kg	74.94	69.44[b]	68.75[b]
SEM	1.76	1.43	1.21
P-value	0.112	0.031	0.049
OM			
60 kg	72.55	74.17[a]	71.13[a]
25 kg	74.05	69.14[b]	68.36[b]
SEM	1.26	1.42	1.01
P-value	0.072	0.013	0.024
EE			
60 kg	71.55[b]	57.38[b]	94.32[a]
25 kg	86.32[a]	79.94[a]	69.66[b]
SEM	3.62	3.87	4.01
P-value	0.016	0.013	0.001
SDF			
60 kg	9.15	25.48[a]	48.94
25 kg	10.22	18.25[b]	39.55
SEM	4.35	2.52	3.66
P-value	0.935	0.041	0.064
Hemicellulose			
60 kg	13.15[b]	45.48[a]	45.35[a]
25 kg	25.06[a]	32.40[b]	32.71[b]
SEM	3.02	4.91	2.76
P-value	0.022	0.034	0.026

[a] Data represent least square means (n=6), and individual pig was treated as the experimental unit. The variant alphabetical superscript in the same column within each diet indicate significant difference at $p<0.05$. CB, corn bran; SBP, sugar beet pulp; SEM, standard error of the mean; SH, soybean hulls.
